# Efficacy Coefficients Determined Using Nail Permeability and Antifungal Activity in Keratin-Containing Media Are Useful for Predicting Clinical Efficacies of Topical Drugs for Onychomycosis

**DOI:** 10.1371/journal.pone.0159661

**Published:** 2016-07-21

**Authors:** Yoshiki Matsuda, Keita Sugiura, Takashi Hashimoto, Akane Ueda, Yoshihiro Konno, Yoshiyuki Tatsumi

**Affiliations:** Drug Research Center, Kaken Pharmaceutical Co., Ltd., Kyoto, Japan; Louisiana State University, UNITED STATES

## Abstract

Onychomycosis is difficult to treat topically due to the deep location of the infection under the densely keratinized nail plate. In order to obtain an *in vitro* index that is relevant to the clinical efficacy of topical anti-onychomycosis drugs, we profiled five topical drugs: amorolfine, ciclopirox, efinaconazole, luliconazole, and terbinafine, for their nail permeabilities, keratin affinities, and anti-dermatophytic activities in the presence of keratin. Efinaconazole and ciclopirox permeated full-thickness human nails more deeply than luliconazole. Amorolfine and terbinafine did not show any detectable permeation. The free-drug concentration of efinaconazole in a 5% human nail keratin suspension was 24.9%, which was significantly higher than those of the other drugs (1.1–3.9%). Additionally, efinaconazole was released from human nail keratin at a greater proportion than the other drugs. The MICs of the five drugs for *Trichophyton rubrum* were determined at various concentrations of keratin (0–20%) in RPMI 1640 medium. The MICs of ciclopirox were not affected by keratin, whereas those of efinaconazole were slightly increased and those of luliconazole and terbinafine were markedly increased in the presence of 20% keratin. Efficacy coefficients were calculated using the nail permeation flux and MIC in media without or with keratin. Efinaconazole showed the highest efficacy coefficient, which was determined using MIC in media with keratin. The order of efficacy coefficients determined using MIC in keratin-containing media rather than keratin-free media was consistent with that of complete cure rates in previously reported clinical trials. The present study revealed that efficacy coefficients determined using MIC in keratin-containing media are useful for predicting the clinical efficacies of topical drugs. In order to be more effective, topical drugs have to possess higher efficacy coefficients.

## Introduction

Onychomycosis is a chronic superficial mycosis of the nails that is difficult to treat. Its prevalence is reported to be 23% across Europe [1], 13.8% in North America [2] and approximately 10% in Japan [3]. Patients with onychomycosis develop physical and psychological issues, including pain, difficulty wearing shoes, secondary infection, and difficulties performing everyday functions due to the resulting nail dystrophy or unacceptable cosmetic appearance [4].

The current preferred therapy for onychomycosis is the oral antifungals, terbinafine and itraconazole. However, their use is limited by hepatotoxicity and drug-drug interactions (especially for itraconazole), which represent a safety concern, particularly in elderly patients, in whom underlying diseases and polypharmacy are common. Routine liver function testing is recommended for patients being treated with oral terbinafine and itraconazole, and their use is contraindicated in patients with impaired liver function [5]. Due to its localized effects, topical drug delivery is desirable for treating nail disorders, which results in minimal adverse systemic events and possibly improved adherence [6]. Thus, safe and efficacious topical therapies against onychomycosis remain an unmet medical need.

Distal lateral subungual onychomycosis (DLSO) is the most common form of fungal nail infection. *Trichophyton rubrum*, the most frequent causative fungus, invades the nail bed under the nail plate in DLSO. Therefore, topical antifungal drugs need to permeate through the nail plate to the nail bed in order to be effective.

The nail plate is composed of keratin proteins that are held together by a disulfide linkage. The nail’s unique properties, particularly its thickness and relatively compact construction, make it a formidable barrier to the permeation of topically applied agents. Moreover, the binding of the drug to keratin in the nail plate reduces the free (active) drug, thereby diminishing the concentration gradient and limiting permeation into deeper tissues [7]. Furthermore, keratin binding would reduce the antifungal potency of a drug in the nail bed. Therefore, the concentration of a topically applied drug fails to reach a therapeutically effective concentration in the nail bed, in which fungi reside.

To date, the number of approved topical anti-onychomycosis drugs is markedly lower than topical anti-tinea pedis drugs. In the topical treatment of onychomycosis, 5% amorolfine nail lacquer (not approved in North America) and 8% ciclopirox nail lacquer have been widely used in Europe and North America, 10% efinaconazole solution was recently launched in North America, Canada, and Japan [8], 5% tavaborole solution was recently launched in North America [9], and 5% luliconazole solution was launched in Japan in April 2016 [10]. Terbinafine formulations are currently being developed for the additional indication of a topical anti-onychomycosis drug [11, 12].

The *in vitro* activities of the above antifungals (excluding luliconazole) have been investigated in media containing a keratin powder concentration of 5% [13, 14, 15]. However, since the nail plate and nail bed have higher keratin concentrations (approximately 80–90%) [16], further studies at higher keratin concentrations than those examined in previous studies are needed in order to more accurately predict the potency of antifungal activity in nails.

We previously reported that efinaconazole permeated the human nail more deeply than amorolfine and to a similar extent to ciclopirox using commercial products with different vehicle compositions [17]. It is important to assess the nail permeation of active pharmaceutical ingredients using the same vehicle composition in order to accurately compare nail permeation between drugs. Furthermore, comparative studies have yet to be conducted on the nail permeation of efinaconazole, luliconazole, and terbinafine.

In an attempt to clarify the favorable properties of topical anti-onychomycosis drugs, we herein evaluated five topical drugs with different physical properties (Table 1) for their *in vitro* human nail permeabilities, *in vitro* human keratin affinity, and *in vitro* anti-dermatophytic activities in the presence of keratin at various concentrations. The aim of the present study is to establish an *in vitro* index that is relevant to clinical efficacy by measuring nail permeation flux and MIC in media without or with keratin. This index will provide a strategy to develop more effective topical anti-onychomycosis drugs in the future.

**Table 1:** Physical properties of five test drugs.

	Drugs				
	Amorolfine	Ciclopirox	Efinaconazole	Luliconazole	Terbinafine
Molecular weight	318	207	348	354	291
ClogP	6.44	2.03	2.15	3.49	5.96

ClogP is the logP calculated using ChemBioDraw Ultra software (version 12.0).

## Materials and Methods

### Antifungals, fungal strains, media, and keratin

Amorolfine hydrochloride and terbinafine hydrochloride were purchased from Tokyo Chemical Industry Co., Ltd. (Tokyo, Japan). Ciclopirox was purchased from Sigma-Aldrich Corporation (St. Louis, MO, USA). Efinaconazole was synthesized at Kaken Pharmaceutical Co., Ltd. (Tokyo, Japan). Luliconazole was purchased from Toronto Research Chemicals (Toronto, Canada).

*T*. *rubrum* NBRC 6203 and *T*. *rubrum* (IFM 46615, IFM 47622, IFM 47627, IFM 46157, and IFM 46204) were obtained from the Biological Resource Center, National Institute of Technology and Evaluation (Tokyo, Japan) and Medical Mycology Research Center, Chiba University (Chiba, Japan), respectively.

RPMI 1640 medium (Nissui Seiyaku, Tokyo, Japan) buffered with 0.165 M morpholinopropanesulphonic acid (MOPS) at pH 7.0 was used in drug susceptibility tests.

Human nails were purchased from Science Care (Phoenix, AZ, USA), which was accredited by the American Association of Tissue Banks. These were powdered using a miller (Wonder Blender, Osaka Chemical Co. Ltd., Osaka, Japan) and defatted with a mixture of ethanol-diethyl ether (1:1, vol/vol). Defatted keratin powder was also made from porcine hooves (O.C. farm, Okayama, Japan) using a similar method to that for human nail keratin powder.

### Affinity of drugs to keratin

The affinities of the test drugs to human nail keratin were determined as described previously [17], with a slight modification. In order to examine the relationship between drug-keratin affinity and increases in the rate of MIC with the addition of keratin to medium, dimethyl sulfoxide (DMSO), which is the same solvent in the two tests, was used. We confirmed that the final concentration of DMSO (1%) did not affect drug-keratin affinity. One hundred microliters of the drug solution in DMSO (50 μg/mL amorolfine, ciclopirox, efinaconazole, luliconazole, or terbinafine) was mixed with 9.9 mL of 0.2 mol/L Tris-HCl buffer (pH 7.4) containing 0.5 g of defatted keratin powder. After shaking at 37°C for 1 h (75 rpm), the suspension was centrifuged. The drug concentration in the supernatant was determined by liquid chromatography-tandem mass spectrometry (LC-MS/MS) (TSQ Quantum Ultra; Thermo Fisher Scientific, Inc., Waltham, MA, USA). The lower limit of quantification by the assay was 0.5 ng/mL for amorolfine, ciclopirox, and efinaconazole and 1 ng/mL for luliconazole and terbinafine. The percentage of the free drug was calculated for each drug, and mean values were compared by Tukey’s multiple-comparison test (EXSUS version 8.0.0; CAC Exicare Corporation). *P* values of less than 0.05 were regarded as significant. The keratin pellet (containing bound drug) was subsequently resuspended in 10 mL of 0.2 mol/L Tris-HCl buffer and washed by shaking for 10 min. The suspension was centrifuged and the washing procedure repeated 5 times. All procedures were performed in triplicate on the same day. The drug concentration of the supernatant obtained after each wash was measured by LC-MS/MS, and the cumulative percent release of the drug was calculated. The cumulative release of each drug after 5 washes was compared using Tukey’s multiple-comparison test. *P* values of less than 0.05 were regarded as significant.

### *In vitro* nail permeation of drugs

Healthy human nails at full thickness were purchased from Science Care (AZ, USA) and cut into squares of approximately 49 mm^2^. The nails were firmly fixed between two fluoro rubbers with a 5-mm diameter hole and mounted in Franz diffusion cells. The fluoro rubbers were used to prevent the leakage of the drug solutions. The receptor compartment was filled with phosphate-buffered saline (PBS) (pH 7.4) containing 4% (wt/vol) bovine serum albumin and 0.01% (wt/vol) sodium azide and placed in an incubator at 32°C. Twelve microliters of cocktail solution (propylene glycol: ethanol = 1:4, vol/vol) including amorolfine, ciclopirox, efinaconazole, luliconazole, and terbinafine at 5% was then singly applied to the dorsal nail surface in the donor compartment. The donor compartment in Franz cells was not occluded. The receptor solution was continuously stirred using a spinning magnetic bar. The receptor fluid was sampled once daily for 18 days, drug concentrations were determined by LC-MS/MS, and the flux (ng/cm^2^/day) was calculated from the cumulative drug amount permeated per nail.

### Effects of keratin concentrations on MIC for *T*. *rubrum*

Two hundred and fifty microliters of MOPS buffered RPMI 1640 with 2-fold serial dilutions of the test drugs was added to the glass tubes without or with 6.25 mg, 25 mg, or 100 mg defatted porcine hoof keratin powder. Two hundred and fifty microliters of MOPS buffered RPMI 1640 containing *T*. *rubrum* microconidia (final fungal concentration: 1×10^4^ cells) was then added (Final keratin concentrations: 0, 1.25, 5 and 20%). It was not possible to set keratin concentrations to greater than 20% because keratin powder at such high concentrations absorbs the medium and forms clumps, thereby making it difficult to mix microconidia and the drugs to be tested. The glass tubes were incubated at 35°C for 7 days. After the incubation, the minimal inhibitory concentration (MIC) was read as the lowest concentration that prevented any discernible growth (100% inhibition).

### Efficacy coefficient for the onychomycosis treatment

Mertin and Lippold [18] introduced an efficacy coefficient to maximize the therapeutic effectiveness of antifungals. This simple equation allows for the estimation and comparison of relative efficacies among various antifungal agents. The efficacy coefficient was calculated by the ratio of the flux of an antifungal drug through the nail plate to the MIC at each keratin concentration (Equation: Efficacy coefficient = flux/MIC).

## Results

### Affinity of drugs to keratin

**Fig 1:**
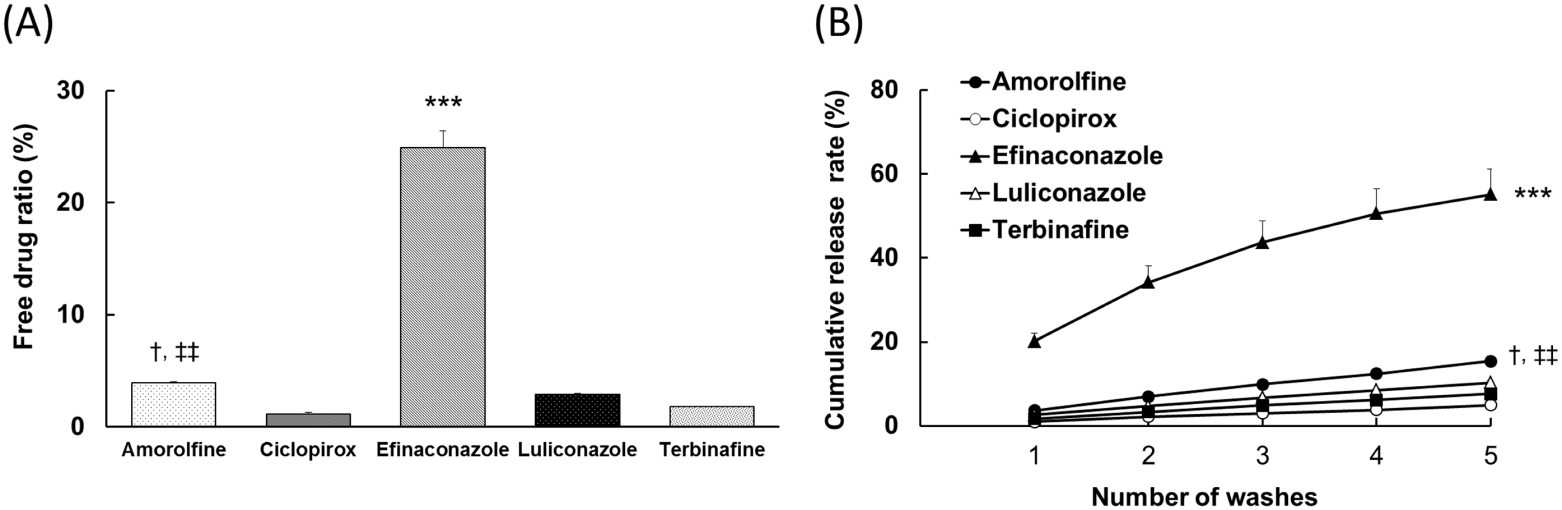
Affinities of five test drugs to keratin. (a) The percentage of the free drug in buffer after shaking at 37°C for 1 h. The graphs represent the mean + SD of three replicates. Tukey’s multiple-comparison test: ***, *P* < 0.001 versus amorolfine, ciclopirox, luliconazole and terbinafine; †, *P* < 0.05 versus terbinafine; ‡‡, *P* < 0.01 versus ciclopirox. (b) The cumulative release of amorolfine, efinaconazole, luliconazole, and terbinafine. Each plot and bar represent the mean + SD of three replicates. Tukey’s multiple-comparison test: ***, *P* < 0.001 versus amorolfine, ciclopirox, luliconazole and terbinafine; †, *P* < 0.05 versus terbinafine; ‡‡, *P* < 0.01 versus ciclopirox.

Amorolfine, ciclopirox, efinaconazole, luliconazole, and terbinafine free-drug levels after their incubation in 5% human nail keratin suspensions were 3.9 ± 0.1%, 1.1 ± 0.2%, 24.9 ± 1.5%, 2.9 ± 0.1%, and 1.8 ± 0.0% (mean ± standard deviation [SD]), respectively. The cumulative drug release levels after five washes were 15.4 ± 1.2%, 5.0 ± 0.6%, 55.1 ± 6.1%, 10.3 ± 0.5%, and 7.6 ± 0.5% (mean ± SD), respectively (Fig 1). The efinaconazole free-drug ratio and keratin release levels were markedly higher than those of the other drugs. The amorolfine free-drug ratio and keratin release levels were slightly higher than those of ciclopirox and terbinafine.

### *In vitro* nail permeation of drugs

The human nail permeation of drugs was investigated in Franz diffusion cells after a single topical application of the cocktail solution including amorolfine, ciclopirox, efinaconazole, luliconazole, and terbinafine at 5% to human nails for 18 days (n = 6). Efinaconazole was detected in the receptor fluids as early as day 2, whereas ciclopirox and luliconazole were first detected on day 4. On day 18, drug concentrations were detected in the receptor fluids of 6/6 nails for efinaconazole, 3/6 nails for ciclopirox, and 2/6 nails for luliconazole. On the other hand, neither amorolfine nor terbinafine was detectable in any receptor fluids. The cumulative permeated amounts of ciclopirox, efinaconazole, and luliconazole on day 18 were 0.221 ± 0.464, 0.351 ± 0.369, and 0.0131 ± 0.0238 μg/cm^2^, respectively. In addition, the fluxes of ciclopirox, efinaconazole, and luliconazole were 31.7 ± 59.2, 38.8 ± 36.0, and 2.23 ± 3.89 ng/cm^2^/day, respectively (Table 2). The order of nail permeability of the test drugs remained unchanged in all human nail used. These results clearly indicated that efinaconazole and ciclopirox permeated through the human nail more deeply than the other drugs.

**Table 2:** Cumulative drug amount permeated after a single application of five test drugs to human nails.

Time (day)	Cumulative amount (μg/cm^2^)				
	Amorolfine	Ciclopirox	Efinaconazole	Luliconazole	Terbinafine
1	NC	NC	NC	NC	NC
2	NC	NC	0.00130 ± 0.00318	NC	NC
3	NC	NC	0.00577 ± 0.01413	NC	NC
4	NC	0.00161 ± 0.00394	0.0127 ± 0.0275	NC	NC
7	NC	0.00420 ± 0.01029	0.0222 ± 0.0389	NC	NC
8	NC	0.0118 ± 0.0288	0.0396 ± 0.0602	NC	NC
9	NC	0.0438 ± 0.1074	0.0817 ± 0.1222	NC	NC
10	NC	0.0590 ± 0.1445	0.107 ± 0.158	0.00163 ± 0.00398	NC
11	NC	0.0783 ± 0.1866	0.140 ± 0.186	0.00252 ± 0.00616	NC
14	NC	0.100 ± 0.233	0.182 ± 0.205	0.00327 ± 0.00800	NC
15	NC	0.130 ± 0.297	0.235 ± 0.262	0.00668 ± 0.01282	NC
16	NC	0.145 ± 0.326	0.258 ± 0.276	0.00745 ± 0.01376	NC
17	NC	0.191 ± 0.414	0.300 ± 0.309	0.0106 ± 0.0194	NC
18	NC	0.221 ± 0.464	0.351 ± 0.369	0.0131 ± 0.0238	NC
Flux (ng/cm^2^/day)	NC	31.7 ± 59.2	38.8 ± 36.0	2.23 ± 3.89	NC

Data represent the mean ± SD (n = 6).

NC, not calculated because the drug concentration in the receptor fluid was below the lower limit of quantification in all samples.

Flux was calculated from the cumulative amount on days 15, 16, 17, and 18.

NC entered as zero in calculations.

### Effects of keratin concentrations on MIC for *T*. *rubrum*

**Fig 2:**
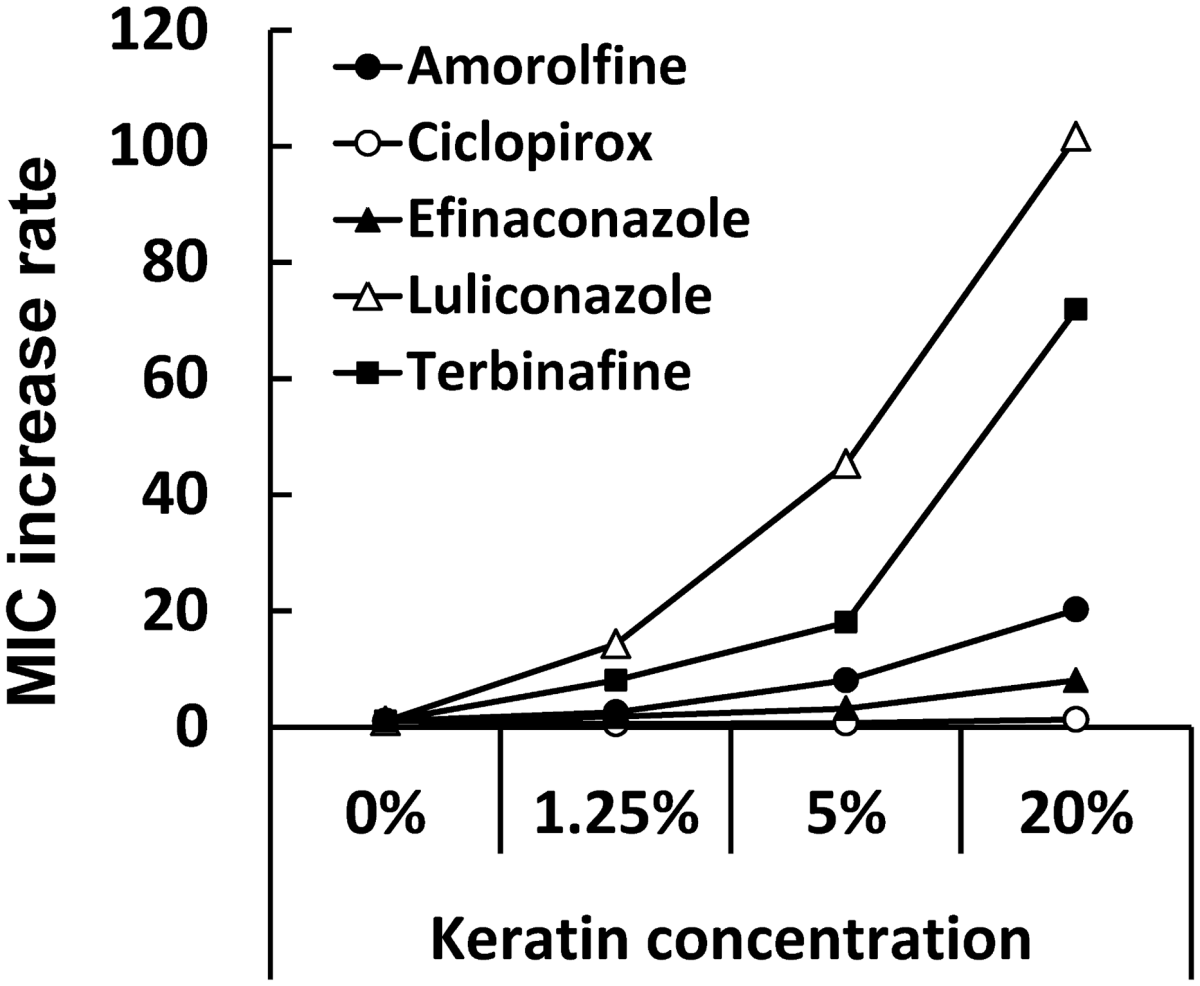
MIC increase rates of five test drugs for *T*. *rubrum* by the addition of keratin. Each plot represents the rates of geometric mean MIC increases (n = 6) to the MIC in medium without keratin.

The influence of keratin concentrations (0, 1.25, 5, or 20%) on the anti-*T*. *rubrum* activities of the five drugs was investigated and compared. The geometric mean MICs of ciclopirox were almost unaltered at all keratin concentrations, but reached the highest values (16–40 μg/mL) among the drugs tested. In contrast, the geometric mean MICs of the four other drugs increased with elevations in the concentration of keratin (Table 3 and Fig 2). The geometric mean MICs in the presence of 20% keratin of amorolfine, efinaconazole, terbinafine, and luliconazole were 20-fold, 8-fold, 72-fold, and 102-fold higher, respectively, than those under the keratin-free conditions. Consequently, the geometric mean MICs under the 20% keratin condition of luliconazole (0.11 μg/mL), efinaconazole (0.11 μg/mL), and terbinafine (0.20 μg/mL) indicated almost the same values, while luliconazole showed the lowest geometric mean MIC (0.0011 μg/mL) under keratin-free conditions. Amorolfine was less active than efinaconazole, luliconazole, and terbinafine at all keratin concentrations.

**Table 3:** MICs of five test drugs for *T*. *rubrum* in medium with and without keratin.

Drugs	Geometric mean MIC (μg/mL) for *T*. *rubrum* in the following keratin concentrations			
	0%	1.25%	5%	20%
Amorolfine	0.039	0.099	0.31	0.79
Ciclopirox	32	16	20	40
Efinaconazole	0.014	0.025	0.044	0.11
Luliconazole	0.0011	0.016	0.050	0.11
Terbinafine	0.0028	0.022	0.050	0.20

Data represent the geometric mean MIC for 6 *T*. *rubrum* strains.

### Efficacy coefficient for the onychomycosis treatment

Since amorolfine and terbinafine were not detectable in any receptor fluids of Franz diffusion cells, it was not possible to calculate their efficacy coefficients. Thus, the efficacy coefficients of efinaconazole, ciclopirox, and luliconazole were calculated (Table 4). The efficacy coefficients of ciclopirox were markedly lower than those of efinaconazole and luliconazole at all keratin concentrations. Although the efficacy coefficients of efinaconazole and luliconazole were almost the same when calculated using MIC in keratin-free media, that of efinaconazole was approximately 10- to 20-fold higher than that of luliconazole when calculated using MIC in the presence of keratin.

**Table 4:** Efficacy coefficients of five test drugs determined using MICs in medium with/without keratin.

Drugs	Efficacy coefficient in the following keratin concentrations			
	0%	1.25%	5%	20%
Amorolfine	NC	NC	NC	NC
Ciclopirox	1	2	2	1
Efinaconazole	2787	1564	878	348
Luliconazole	2034	143	45	20
Terbinafine	NC	NC	NC	NC

NC was not calculated because it was not possible to determine the fluxes of the two drugs.

## Discussion

To date, difficulties have been associated with proposing a strategy to develop more effective topical anti-onychomycosis drugs because a method for predicting clinical efficacy has not yet been fully established. Mertin and Lippold [18] introduced an efficacy coefficient determined using nail permeability (flux) and antifungal activity (MIC) in keratin-free medium in order to predict the therapeutic effectiveness of antifungals. However, the relationship between efficacy coefficients and clinical efficacies remains unclear. In the present study, in order to establish an *in vitro* index that is relevant to clinical efficacy, we profiled five topical antifungal drugs using a novel system that directly compares the human nail permeabilities of antifungal drugs under physiological conditions and an *in vitro* anti-dermatophytic activity assay system using keratin-containing media.

It is considered important for topical anti-onychomycosis drugs to have high permeabilities in the human nail and exhibit antifungal activities in the nail bed. Several antifungal compounds have been investigated for their human nail permeabilities using various diffusion cells [19]. However, there are difficulties associated with correctly comparing nail permeabilities between antifungal drugs because human nails exhibit marked individual differences, e.g., in thickness and uniformity. Indeed, we experienced that a certain compound that highly permeates one human nail sometimes does not as easily permeate another, even if they have the same appearance. As a consequence, the results vary depending on nails used, thereby making difficult to directly compare nail permeation between compounds. Therefore, it is important to compare permeation of compounds by using a single nail at a time. Thus, in the present study, we investigated nail permeation using a cocktail solution of the five test drugs at the same time and confirmed that the order of nail permeability of the test drugs remained unchanged in any single human nail used, which indicates that our assay system enables accurate comparisons of drug permeabilities even in nails obtained from various donors. We previously evaluated the nail permeabilities of a large number of compounds using a cocktail solution and confirmed the order of nail permeation remained unchanged in our background data.

Previous studies have examined the human nail permeation of amorolfine, terbinafine, and luliconazole [18, 20, 21]. A receptor fluid containing ethanol or methanol at 42–60% was used in these studies to detect drug nail permeation. Drug permeation was greater than that observed in this study. A comparison of nail drug permeabilities in the previous studies and the present study is not reasonable because alcohol increases the distribution of test drugs to the receptor fluid. In the present study, considering the physiological condition of the nail bed, we used alcohol-free solution as the receptor cell fluid and compared the nail permeabilities of the test drugs. In this system, ciclopirox and efinaconazole permeated the human nail plate deeply, whereas the permeabilities of amorolfine, luliconazole, and terbinafine were extremely low. The order of the nail permeation of amorolfine, ciclopirox, and efinaconazole was consistent with that of our previous findings using commercial products [17].

From a physiochemical viewpoint, nails behave more like a hydrophilic gel membrane as opposed to a lipophilic membrane, such as the stratum corneum [22]. Drug permeation into the nail is influenced by the physicochemical properties of the drug (e.g., hydrophilicity and molecular weight). The water solubility of efinaconazole (29 μg/mL, unpublished data) is higher than those of the other test drugs [23]. Furthermore, the keratin affinity of drugs is also considered to be an important factor affecting nail permeability [6, 18]. The present study demonstrated that efinaconazole showed a lower affinity to human nail keratin than the other drugs. Taken together, these results indicate that the high human nail permeation of efinaconazole is due to two favorable physicochemical properties, its lower keratin affinity and higher hydrophilicity than those of the other drugs. Ciclopirox permeated the human nail in spite of its high keratin affinity. This result may be explained by it having the lowest molecular weight (207) among the drugs tested.

Although the keratin affinity of luliconazole was similar to those of amorolfine and terbinafine, its nail permeation flux was higher. Several previous studies have shown that the drug permeation flux correlates with the degree of drug saturation in a formulation [24, 25]. The five drugs tested in the present study had different solubilities in the vehicle (propylene glycol: ethanol = 1:4, vol/vol) used in the human nail permeation study (approximate maximum solubility; amorolfine: 30%, ciclopirox: 20%, efinaconazole: 50%, luliconazole: 8%, and terbinafine: 40%) (unpublished data). Among the drugs tested, the maximal solubility of luliconazole was close to a concentration of 5% in the present study. Therefore, the high degree of saturation of luliconazole may have partly contributed to its higher nail permeation flux than those of amorolfine and terbinafine.

Interpreting our results from a lipophilicity (ClogP: calculated octanol/water partition coefficient) viewpoint, efinaconazole had lower ClogP (2.15) than those of amorolfine, terbinafine, and luliconazole (ClogP: 6.44, 5.96, and 3.49, respectively), as described in Table 1. Hansen et al. previously reported a direct relationship between the binding rates of several drugs to keratins and logP (octanol/water partition coefficient) [26]. The lower keratin affinity of efinaconazole is considered to be partly due to its lower lipophilicity than those of the three drugs. This low keratin affinity may contribute to a high concentration of the free drug and its concentration gradient in the nail, resulting in permeation to the deeper layers and nail bed. Although ciclopirox has the lowest ClogP (2.03), it showed high keratin affinity. Ciclopirox chelates trivalent cations, such as Fe^3+^ and Al^3+^ [27], unlike the four other drugs. Therefore, its high keratin affinity may be due to binding to the protein and trivalent cations included in keratin powder.

Furthermore, permeated antifungals must retain their antifungal activities in the nail bed in which dermatophyte resides. However, many antifungal agents are inactivated in the nail bed by their high affinities to keratin [13]. Therefore, in order to predict the potency of antifungal activity in the nail bed, the *in vitro* activities of the five test drugs were determined under various keratin concentrations. Conversely, the drug susceptibility of dermatophytes is commonly evaluated according to the CLSI M38-A2 method using keratin-free RPMI 1640 medium [28]. The MICs of amorolfine, efinaconazole, luliconazole, and terbinafine increased with elevations in keratin concentrations (0, 1.25, 5, and 20%) in RPMI 1640 medium. In the presence of 20% keratin, the rates of MIC increases for luliconazole, terbinafine, amorolfine, and efinaconazole were 102, 72, 20, and 8, respectively. The rates of increases in MIC were largely associated with the degree of keratin affinity. The high rates of MIC increases for luliconazole and terbinafine were presumably due to their high keratin affinities. Since the concentration of keratin in the nail is approximately 80–90% [16], the antifungal activities of luliconazole and terbinafine would be further reduced in the infection site. Luliconazole was the most active in keratin-free RPMI 1640 medium, as previously reported by Koga et al. [29]. However, luliconazole was as active as efinaconazole and terbinafine in the presence of 20% keratin.

Ciclopirox showed high affinity to keratin. Nevertheless, its antifungal activity was not affected by keratin concentrations. The geometric mean MICs (16–40 μg/mL) of ciclopirox in the presence of keratin were 200- to 1000-fold higher than those of the four other drugs. The ratios of the MICs of ciclopirox to keratin were 8- to 320-fold higher than that in the keratin affinity test. We confirmed that ciclopirox showed a high free-drug ratio in the keratin affinity test at a high ratio of the drug to keratin (unpublished data) presumably because of the saturation of drug-keratin binding, and, thus, it was considered that the activity of ciclopirox was not influenced by keratin.

As described above, the success of topical therapy for onychomycosis depends on whether the permeated drug amount in the deep nail bed is retained above its MIC. In order to predict clinical efficacy, the efficacy coefficients of several antifungals have been calculated by an equation of flux/MIC determined using keratin-free medium [10, 18, 30]. However, the binding of antifungals to keratin in nail bed reduces the availability of active (free) drugs and markedly weakens their antifungal potencies. Therefore, we introduced that the efficacy coefficient be determined using MIC values obtained in the presence of keratin.

The results of the present study suggest that amorolfine, ciclopirox, and terbinafine have lower efficacy coefficients than efinaconazole and luliconazole. In clinical trials with 5% amorolfine lacquer, 8% ciclopirox lacquer, and 10% terbinafine solution, complete cure rates were low (0.96%, 5.5–8.5%, and 1.2%, respectively) [31, 32]. In contrast, in clinical trials with 10% efinaconazole solution and 5% luliconazole solution, complete cure rates in Japanese patients were high (28.8% and 14.9%, respectively) [33, 34]. The order of complete cure rates was consistent with that of efficacy coefficients determined using MIC in keratin-containing media rather than that in keratin-free media. Therefore, the efficacy coefficient determined using MIC in keratin-containing media should be useful for the prediction of clinical efficacy.

In conclusion, the present study revealed that the efficacy coefficient determined using MIC in keratin-containing media is useful for predicting the clinical efficacies of topical anti-onychomycosis drugs. Topical drugs have to possess higher efficacy coefficients to be more effective. In order to achieve this, not only strong antifungal potency, but also the favorable physicochemical properties of low keratin affinity along with a low molecular weight need to be carefully considered in the future design of topical anti-onychomycosis drugs.
